# Households, fluidity, and HIV service delivery in Zambia and South Africa – an exploratory analysis of longitudinal qualitative data from the HPTN 071 (PopART) trial

**DOI:** 10.1002/jia2.25135

**Published:** 2018-07-19

**Authors:** Graeme Hoddinott, Hanlie Myburgh, Laing de Villiers, Rhoda Ndubani, Jabulile Mantantana, Angelique Thomas, Madalitso Mbewe, Helen Ayles, Peter Bock, Janet Seeley, Kwame Shanaube, James Hargreaves, Virginia Bond, Lindsey Reynolds, the HPTN 071 (PopART) Study Team

**Affiliations:** ^1^ Department of Paediatrics and Child Health Faculty of Medicine Desmond Tutu TB Centre Stellenbosch University Stellenbosch South Africa; ^2^ Zambart, School of Medicine Lusaka Zambia; ^3^ Department of Clinical Research London School of Hygiene and Tropical Medicine London UK; ^4^ Department of Global Health and Development Faculty of Public Health and Policy London School of Hygiene and Tropical Medicine London UK; ^5^ Department of Social and Environmental Health Research Centre for Evaluation London School of Hygiene and Tropical Medicine London UK; ^6^ Department of Sociology and Social Anthropology Faculty of Arts and Social Sciences Stellenbosch University Stellenbosch South Africa; ^7^ Population Studies and Training Center Brown University Providence RI USA

**Keywords:** HIV testing, antiretroviral therapy (ART), adherence, southern Africa, mobility, household residence, membership

## Abstract

**Introduction:**

Population distributions, family and household compositions, and people's sense of belonging and social stability in southern Africa have been shaped by tumultuous, continuing large‐scale historical disruptions. As a result, many people experience high levels of geographic and social fluidity, which intersect with individual and population‐level migration patterns. We describe the complexities of household fluidity and HIV service access in South Africa and Zambia to explore implications for health systems and service delivery in contexts of high household fluidity.

**Methods:**

HPTN 071 (PopART) is a three‐arm cluster randomized controlled trial implemented in 21 peri‐urban study communities in Zambia and South Africa between 2013 and 2018. A qualitative cohort nested in the trial included 148 purposively sampled households. Data collection was informed by ethnographic and participatory research principles. The analysis process was reflexive and findings are descriptive narrative summaries of emergent ideas.

**Results:**

Households in southern Africa are extremely fluid, with people having a tenuous sense of security in their social networks. This fluidity intersects with high individual and population mobility. To characterize fluidity, we describe thematic patterns of household membership and residence. We also identify reasons people give for moving around and shifting social ties, including economic survival, fostering interpersonal relationships, participating in cultural, traditional, religious, or familial gatherings, being institutionalized, and maintaining patterns of substance use. High fluidity disrupted HIV service access for some participants. Despite these challenges, many participants were able to regularly access HIV testing services and participants living with HIV were especially resourceful in maintaining continuity of antiretroviral therapy (ART). We identify three key features of health service interactions that facilitated care continuity: disclosure to family members, understanding attitudes among health services staff including flexibility to accommodate clients’ transient pressures, and participants’ agency in ART‐related decisions.

**Conclusions:**

Choices made to manage one's experiential sense of household fluidity are intentional responses to livelihood and social support constraints. To enhance retention in care for people living with HIV, policy makers and service providers should focus on creating responsive, flexible health service delivery systems designed to accommodate many shifts in client circumstances.

## 1 Introduction

An estimated 65% of people living with HIV (PLHIV) in Zambia and 56% in South Africa 1 are currently on antiretroviral therapy (ART). Both countries have committed to scaling up their ART programmes to all PLHIV 2, 3. However, multiple social, historical, economic, and health service challenges make this aim difficult to achieve 4. Optimal implementation of ART programmes requires predictable progression of clients through the sequential steps of the HIV care continuum 5, 6. In southern Africa, high levels of population mobility and what we term “household fluidity” create significant challenges for stability along the care continuum 7, 8. The concept of household fluidity emerges from anthropological studies that recognize the “problem of domestic group pliancy and labile household compositions … where both individuals and households seemed to move about almost continuously” 9. Households are not limited to one geographic location or social network. Social networks are often geographically diffuse. We define household fluidity as an aggregate property of the person's experiential sense of their social networks’ security, stability and reliability. This experience is on a continuum that ranges from stasis to chaotic unpredictability. The person's sense of household fluidity manifests in their experiences of belonging in a space with contemporary and historical dynamics and places parameters on their identity maintenance in that space. People seek a balance of stimulation/novelty/unpredictability to stability/routine/security in their social networks. The experience of household fluidity can therefore prompt mobility as people move towards their optimum balance. In addition, mobility causes changes in the experience of household fluidity both by imposing geographic barriers and by changing the interpersonal dynamics between household members. While individual and population mobility are active processes, we suggest that household fluidity is an inherent characteristic of everyday life that requires constant management.

Population distributions, family and household compositions, and people's sense of belonging and social stability in southern Africa have been shaped by tumultuous, interwoven, and continuing large‐scale historical disruptions at multiple levels of political and social geography 10, 11, 12. In contemporary Zambia, the country's geographic position – sharing national borders with eight other countries – and its role as a hub for regional trade routes have led to particularly high patterns of mobility. In South Africa, *apartheid*‐era policies formalized iniquitous place boundaries based on racial and ethnic classification and further distorted patterns of mobility and family and household structures 13, 14, 15, 16, 17, 18. These processes have shaped contemporary experiences of social inequity, including unequal burdens of disease and political and social marginalization. Many southern Africans are angry, mistrustful and disenchanted by the unequal distribution of land ownership and socio‐economic opportunities that perpetuate poverty and serve as a reminder of their disempowerment 19, 20.

Demographic categorizations of population mobility prioritize a distinction between long‐term resettlements which change the population denominator, and short‐term in‐ and out‐flows of people through places. Much research has been dedicated to describing and explaining patterns of mobility that recur with cyclical regularity 9, 21, 22, 23, 24, 25. Other research has focused on the influences of household structure and interpersonal politics on individual‐level drivers of intermittent mobility 26, 27, 28, 29, 30. More recently, social scientists have proposed a reframing of mobility to also take account of how mobility intersects with processes of household fluidity and social instability 31, 32, 33, 34. In HIV research, there has also been an increasing focus on mobility highlighting how mobile populations are both at increased risk of HIV infection 35 and are more likely to fall out of the HIV care continuum 36. High rates of mobility amongst particular groups or at specific life stages can create discontinuities along the care continuum. For example, young people, who are a key priority group for HIV service delivery 37, often experience mobility events as they transition from school learner to job seeker. Further, their household composition can change significantly as caregivers move in and out of the home, particularly in households affected by HIV 38, 39. Similarly, sub‐groups within geographic communities – including sex workers 40, truck drivers 41, and people living with disabilities 42 – experience different household mobility patterns, which shape their HIV risk and access to services.

To explore these dynamics, we suggest household fluidity as an analytic lens through which we aim to (1) describe patterns of household membership and residence in 13 study communities in South Africa and Zambia; (2) explore participants’ narratives about why these patterns come about; and (3) describe key features of client/health service relationships that can enable continuity of care in the context of high levels of household fluidity. We discuss how more responsive, context‐specific service delivery models can maintain continuity in the context of fluidity.

## 2 Methods

### 2.1 Study design and period

HPTN 071 (PopART) is a three‐arm cluster randomized controlled trial implemented between 2013 and 2018 in 21 peri‐urban study communities – 12 in Zambia and nine in South Africa 43. Geographically, study communities were defined as the catchment area of a primary health facility. The communities ranged in size from approximately 15,000 to 100,000 adult residents and were typical of high‐burden, low‐resource places in the region. A nested social science evaluation includes a qualitative cohort of households. In Zambia, participants for this cohort were recruited between January and March 2017. In South Africa, some participants were recruited in an exploratory phase between August and December 2015, while the majority were recruited between March and July 2016. The cohort closed in both countries by March 2018.

### 2.2 Sample

In total, the qualitative cohort includes 148 households in Zambia and South Africa recruited in four of the Zambian study communities and all nine South African study communities. The study communities in Zambia were purposively sampled for geographical and study arm representation. Participants were sampled purposively to ensure diversity by trial arm, proximity to local health facility, HIV pathways (including self‐reported HIV status and having tested HIV‐negative or not tested for HIV), age, gender and household structure. In South Africa, the sampling approach also followed the principle of extreme cases to include people at greater risk of HIV acquisition and who are more socially marginalized – including cisgender female sex workers, men who have sex with men, transgender women, people living with disabilities, and young people aged 15 to 24. Finally, participants were sampled to ensure that at least half of the total households included at least one member who self‐reported living with HIV.

### 2.3 Data collection processes

Data collection was informed by ethnographic and participatory research principles. The researchers recorded their discussions with voice recorders and semi‐structured field notes and took pictures of relevant activities. All interactions with participants happened *in situ* in the study communities – usually in their home, but also often as they moved about the study community completing their daily activities. Researchers interacted with households for several hours per interaction and multiple times over the course of the data collection period. Data collection was structured into six modules implemented sequentially but with flexibility to iterate between topics – (1) household, kin, and relational networks, (2) place and space, (3) getting by, (4) sex, love, and romance, (5) HIV service access, and (6) horizons, ambitions, and fears. We draw primarily on the first two of these modules for this analysis, supplemented by our experiences implementing the cohort overall. Participatory research activities in these two modules included: kinship mapping, community mapping, historical narratives of households’ movement into the study community, and timelines of household members’ weekly activities.

### 2.4 Data analysis processes

The data analysis process was reflexive and included routine, structured written reflections by data collectors, monthly analytic workshops of country social science teams, and a targeted review of all primary data by the first five co‐authors. The findings are descriptive narrative summaries of key emergent ideas with illustrative case examples. All interpretations of data were discussed with the country social science teams and expert reviewers as a sense checking mechanism.

### 2.5 Ethical considerations

The trial – including all nested social science – was approved by the London School of Hygiene and Tropical Medicine, University of Zambia, and Stellenbosch University research ethics committees. All participants signed written informed consent per guidance of the in‐country research ethics committee. Household participation was by consensus of all household members. All data are stored securely and reported on using pseudonyms to protect participant confidentiality. Participants did not receive any cash incentive. However, in South Africa research staff had discretionary allowances of approximately 6 USD per day and in Zambia were able to claim back reimbursement for field expenses to contribute to shared meals and other costs of living with participant households.

## 3 Results

### 3.1 Thematic patterns of household membership, residence, and associated fluidity

**Figure 1:**
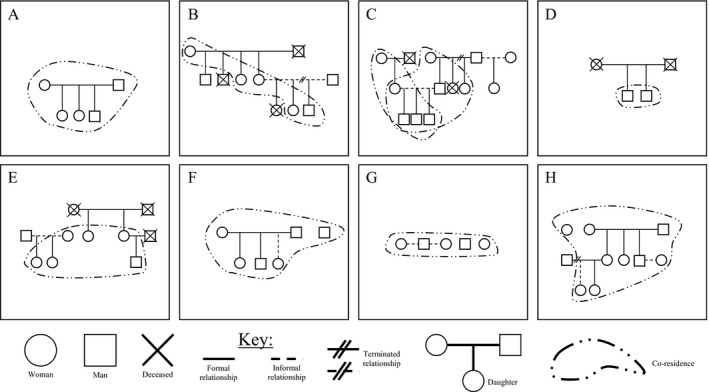
Patterns of household membership and residence.

We identified eight thematic patterns of household membership and residence with implications for fluidity (see Figure 1 as illustrative representations of each pattern). These patterns are changeable and overlapping and should therefore *not* be reified as diagnostic categories. We present them here as cross‐sectional illustrations of diversity in household membership and residence arrangements common in the study communities. Further, they serve as a context within which to understand study community members’ experiences of fluidity.

The simplest pattern of household membership and residence identified in our sample is a stable household consisting of two parents and one or more children (see Figure 1A). Although many participants identify this social grouping (the nuclear family) as defining their most immediate family, very few share consistent, stable residence with all members of their nuclear family in households without other residents. More commonly, household residence is spread across generations (see Figure 1B). These households are generally structured around grandparents – often grandmothers – their adult daughters and their daughters’ children. Many of these households are multi‐nodal, with sets of grandparents that are stably resident in physical structure but children and grandchildren who move between such nodes (see Figure 1C). The proximity of physical structures and household networks can range from being a few doors down the road to neighbouring provinces. There is often an enduring link of kinship or family membership between these places which encourages movement of household members – especially children and younger adults. In contrast, some participants live in very isolated households with limited membership networks, or even sometimes alone (see Figure 1D). These adults – usually men – had often moved recently into the community or from another household in the same community. Residents of these smaller, less stable households are particularly mobile, moving between work and recreational activities like visiting taverns and other social networks.

Participants identified these first four patterns of household residence as extensions of biological ties between family members. Other household patterns illustrate adaptations to these four patterns to include non‐biologically related members. For example, a small group of adult women – who may be biological sisters or cousins, but often are not – cohabitate to pool resources to care for their children (see Figure 1E). Commonly, households with more stable resources foster additional members (see Figure 1F). The household members who are fostered are often new “girlfriends”/wives of young men who remain resident in their parents’ home, children from the extended family or neighbourhood, or people who are chronically ill or living with disabilities (particularly in South Africa where people with disabilities receive a government grant equivalent to 140USD per month). Some households are also arranged to support an intentional survival venture or common goal (see Figure 1G). Often these activities are clandestine (such as selling sex, unlicensed alcohol, or drugs). Lastly, over time households’ membership and residence dynamics change and represent a complex and shifting combination of patterns with internal tensions between members (see Figure 1H).

A household member's fluidity is an outcome of the overall type of household membership and residence pattern of their household(s). For example, households like “A” are generally experienced as more stable than households like “G” (see Figure 1) because familial bonds are often more enduring than enterprise‐associated bonds. Further, households like “G” are experienced as more fluid than “A” because of the contingent and informal nature of associations between members of the household and their other family and social networks outside the residence.

### 3.2 Participants’ explanatory narratives about fluidity

Overall, we found that households are extremely mobile, with various people moving in and out on a daily, monthly and yearly basis. People move within their local neighbourhoods but also across much wider areas of the local town, city, and country. We identified six categories of reasons people provided to explain these movements (see Table 1). This mobility creates dynamic processes where participants spend multiple nights outside of their primary residence over the course of a month. Further, their mobility means that residents consider themselves members of multiple households over the course of a year. The main explanations participants offered for high levels of mobility were economic survival/seeking work or opportunities, seeking shelter and safety, fostering interpersonal – often romantic – relationships, observing cultural, traditional, religious, or familial gatherings and otherwise maintaining a social life, being institutionalized, and maintaining patterns of substance use. Participants pointed out that these dynamics are often very unpredictable as life circumstances change rapidly and new demands for mobility arise.

**Table 1:** Reasons participants give to explain fluidity – with examples

	Examples
Economic survival/seeking work or opportunities	Participants talked about household members that migrated outside the community for work: “My father spends days away from home when he goes for business outside the country.” (Z10^a^_man_17 yrs) J (SA19_woman_43 yrs) lives under a bridge 6.6 km outside of SA19, together with her daughter and son. J and the other residents there go out on Mondays and Wednesdays to “scurry.” They “scratch in the bins for boxes and plastic and things like that” (SA19_woman_25 yrs). Sometimes, they find packages of food intentionally left out by residents of homes. Through these movements, participants reported that they were able to ensure they have enough to eat. Young people, especially in Zambian study communities, mentioned that they are also mobile to support the family business. “I go to sell groundnuts in town,” one woman explained (Z6_woman_17 yrs)
Seeking shelter and safety	F is a transgender woman who sells sex and shares a garage with others behind a house. She explains how her living arrangements have changed over the last few years: “Back then I didn't stay here [in this garage]… I lived there in this road. From there I moved further up to where I lived previously. And then I started sleeping here in the bush, me and another [transgender] girl… and then I met S and I asked her if I could sleep [here in the garage].” (SA14_woman_30 yrs) L is a young man living with HIV from Z6, who was staying with his aunt before he was incarcerated. After his release from prison his aunt asked him to look for a house to rent. He relocated from his aunt's house in a low density area to Z6 (his aunt pays his house rentals). (Z6_man_18 yrs) J is a woman who had recently moved with her family to another part of the community. She explained this as a choice they had to make to escape gangsters who were endangering the community, saying “There we didn't even stay a year, because they broke in a lot.” (SA18_woman_37 yrs)
Fostering inter‐personal – often romantic – relationships	One woman explained that she moved out of her parents’ house to go stay with her boyfriend with whom she had a child because, “We follow that tradition that children don't sleep together with a boyfriend under the roof with the parents.” (SA21_woman_22 yrs) N is a young man living with HIV who has been in his maternal uncle's foster care ever since the death of his parents. He says “growing up, I have never known my biological parents. I was told they died when I was still a baby, I have never seen them…Three of my brothers are not here they live in Zimbabwe with my father's relatives … but I only met them [my brothers] once.” (Z7_man_22 yrs) Another woman described how her social world and physical residence shifted when she separated from her husband, “that time when my ex‐husband and I were separated.” Financial constraints forced her to move: “so then I couldn't afford the rent anymore and had to move.” (SA18_woman_37 yrs)
Observing cultural, traditional, religious, or familial gatherings and maintaining a social life	A young man says that he travels to his parents/extended family's home in a neighbouring province multiple times a year for gatherings and for traditional observances. “In December 2013 I went to Eastern Cape for my circumcision ceremony.” (SA13_man_21 yrs) Young people in Zambia mentioned regularly going to other parts of the community outside their residential area for entertainment: “as for me the places where I'm usually found are X, Y, Z … they are many … except here in K [place of residence] and the reason why I like those places is because there is more entertainment. Here in K you will never see someone mingling around they'd say, ‘ah no, we are not in the ghetto’.” (Z7_woman_17 yrs).
Being institutionalized	J is a 22 year old man who was staying with his girlfriend in a two bedroom wooden shack in SA19. He went to prison in March 2016 and was released a few months later. His girlfriend had started a new relationship and he no longer had a place to stay. A is a 19 year old young woman living with HIV in Z10 who had to relocate (within Z10) to take care of her aunt's house and business while her aunt was ill in hospital for slightly over two months. After her aunt's death, she moved back to her previous home.
Maintaining substance use	Many of the households, especially in South Africa, include household members connected to drug use. These individuals smoke *tik* (methamphetamine) together on a daily basis and build networks around their drug use that involve regular movements to acquire and share drugs. B, his sister, his brother, and his brother's girlfriend, along with friends and acquaintances, all in their late teens and twenties, spend large parts of their day in a derelict house that his father owns. They move around the community looking for things to sell or steal to support their habit. When the research team asks B about his weekly routine he replies “Every day is like our Mondays. Yes, we have our priority and responsibility like that, but the main thing is all about drugs.” (SA19_man_29 yrs)

a Study communities are lettered Z (Zambia) or SA (South Africa), and numbered 1 to 21.

Further, participants described how high mobility creates an abiding sense of fluidity, disturbing their attempts to maintain a sense of belonging and social stability. This sense of fluidity must be understood in the context of an implicit moral responsibility to share socio‐economic support with family and community members. Fulfilling this responsibility was interrelated with how strongly participants felt they belonged in the place and part of the social network. The participants’ sense of their household fluidity related directly to how much they felt they could rely on relationships during times of instability – for example, ending romantic partnerships, shifts in childcare responsibilities, limited economic opportunities, or simply when needing a place to stay. Short‐ and long‐term movements were also oftentimes the result of interpersonal conflict between household members or romantic partners that disturbed the person's household fluidity equilibrium. One example of these dynamics relates to a South African family in which the teenage daughter became pregnant and left school, souring the relationship with her mother. As a result, the daughter now moves between her boyfriend, her grandparents, and her biological father and struggles to manage shifts in her identity – as a girlfriend, grandchild and estranged child – in these spaces. She experiences her household as threateningly fluid (field notes, 27 October 2016; 31 October 2016; 24 November 2016).

South African participants often expressed a general dissatisfaction about life in their communities that informed their sense of not belonging to particular spaces. Some participants considered communities other than where they are resident to be “home.” One example of this is a family who has experienced high levels of crime and violence. The field worker explains, “In times of trouble, the family would relocate to another province. Her family finds solace there. Where they currently live [SA16]^a^ is perceived as a place of suffering” (field notes, 09 November 2016). Participants in Zambia expressed a stronger sense of belonging to their communities, and also usually described them as peaceful. Dissatisfaction was usually expressed towards particular spaces well known for criminal activity, the sale of drugs and alcohol. Friendship networks revolved around neighbours who were mostly described as supportive.

### 3.3 Examples of care continuity in contexts of fluidity – key features

Experiencing one's household as highly fluid made it difficult for many participants to access HIV services consistently. However, in study communities with multiple avenues for HIV testing – including at facilities, in community venues, and door‐to‐door – participants indicated that they were more likely to find at least one option that suited their requirements.

For study participants living with HIV, fluidity created three inter‐related challenges to continued ART access. Firstly, moving between households to manage fluidity meant that physical distance and associated travel costs to health facilities were often changing. Secondly, disruptions to ART continuity as a result of strategies to manage fluidity impacted negatively on PLHIV's relationships with health facility staff and led to experiences of shame related to being labelled as “defaulters.” Thirdly, changes in social networks destabilized fluidity equilibriums and created new challenges related to the management of disclosure, care and support. Despite these challenges, some participants living with HIV are able to maintain ART continuity even in the context of fluidity. The three vignettes summarized in Table 2 illustrate the many challenges PLHIV in highly fluid contexts experience in maintaining ART continuity. The vignettes also demonstrate three key features that enable these participants to overcome these challenges: disclosure to close family members, understanding attitudes and flexibility among health services staff, and participants’ agency to decide on what terms they will be on ART. For N, being on ART means escaping the homophobic restrictions of SA18, for P it is about being a young married man and providing for his young family, and for S it is about looking good to stay economically independent. In each instance, fluidity is not a barrier to their ART continuity. Rather it is just another feature of the context of their lived experiences.

**Table 2:** Case descriptions of care continuity in contexts of fluidity

	Case description
Managing geographic and social distance to access antiretroviral therapy (ART)	N is a gay man who sells sex in SA18. He was diagnosed as living with HIV in 2007 and has been on ART since 2013. N lives in SA18 but he chooses not to access ART at the local health facility <3 km from his home. Instead, he travels 30 km to access ART at a specialized men's health clinic close to Cape Town CBD. N explains his choice with reference to his lack of connection and sense of safety in his community of residence. N prefers not to socialize with other residents of SA18, nor does he work locally. While N enjoys a night out, he does not visit any taverns in SA18 because he is afraid he will be assaulted for his sexual orientation. Despite his decision to access ART far from his place of residence, N has devised various ways to ensure continuous adherence to ART. When he does not have taxi fare, N stows away on the train and walks 40 min from the station to the clinic. N has an arrangement with the clinic to collect his ART early in case he cannot arrive on an appointment date. He also receives additional months’ supplies over the annual festive season so that his ART is not interrupted by travel in December to January for annual holidays in the neighbouring province.
Ensuring treatment continuity in shifting settings	P is a young man who lives alone in Z6. He is married to a 16 year old wife who recently gave birth. Both mother and child are HIV negative. P's wife is living with her mother's family while the baby is young. Both of P's parents are deceased. When he was younger, his parents had him sent to prison for stealing their money and because, he says, “they wanted to teach him a lesson.” P is a trader, travelling to the swamps for months at a time to buy fish and meat to resell in the community. When he is not trading he is drinking at the bar in the market. P learned that he is living with HIV when he was in prison. He started on ART a week later. He says that at the time the health staff were well‐educated and explained the seriousness of missing doses and the importance of eating well. They told him that if he took his medicines and ate appropriately, he would regain lost weight and be healthy. P has only disclosed his status to his wife and close relatives. Now that he is out of prison, he accesses ART at the local clinic. He says he feels safe there because everyone is there for the same reason – accessing ART. When P knows that he will not be available for a subsequent clinic appointment he asks for his ART to be dispensed in advance. When something unplanned arises, his wife is able to collect his ART on his behalf.
Balancing treatment and economic/work‐related requirements through social support	S is a woman who sells sex along a regional road. She used to live with Z in a neighbouring community, but has since moved in with a friend, whom she calls a sister, in SA14. S learnt that she is living with HIV when she was 18 years old after the birth of her child. She has disclosed to her family and to colleagues in different houses where she has worked selling sex. She says a local sex worker advocacy NGO motivated her to start ART in 2015. In 2016, she experienced a treatment interruption because she says the ART caused her to gain weight – implicitly jeopardizing her livelihood. In early 2017 she began taking her ART again alongside an exercise regime to maintain her ideal weight. Her co‐resident friend reminds her to take her ART and she has fostered a positive relationship with health workers at her local health facility who understand the importance of her maintaining her weight.

## 4 Conclusions

We described the complexity of household membership and residence in 13 study communities. We showed the interlinked reasons why choices about managing household fluidity are both ubiquitous and intentional responses. We further explored features of the interactions between clients, their social systems, and health services that facilitate continuity of HIV‐related services access even in contexts of high fluidity. Further, by describing people's household contexts, their reasons for moving, and the continuity of social links even after moving, we are able to begin to explore opportunities to manage the impacts of fluidity and HIV services more efficiently. Health services that are fixed and geographically static create challenges for managing continuity of care in these contexts 44. Some gains have been made through community‐based 45, door‐to‐door 46, 47, and mobile 48 HIV service delivery, but gaps in the stability of the care continuum remain. In contexts of high fluidity, it is the granular adaptability of health services to client needs that enables care continuity. The paper contributes to a growing body of literature that aims to develop a more nuanced conceptualization of household fluidity in the context of HIV 49, 50, 51. This manuscript is about understanding the inherent fluidity in the lives of people living in the highest HIV prevalence region of the world and how this fluidity intersects with their engagements with HIV services. One intersection is that a fixed/static health service system necessarily experiences patient fluidity as a challenge to manage. As illustrated in the vignettes, some participants are able to effectively manage threats to continuity of HIV service access that result from fluidity pressures. These threats include unstable social forms, physical and social distance, HIV and identity‐related stigmas, and the difficulties of maintaining livelihoods in contexts of constraint. However, those who manage to receive consistent quality care do so with difficulty in a system that is not designed to accommodate their often unpredictably fluid life experiences.

It is important to acknowledge that choices around management of household fluidity are often intentional and necessary responses to ensure livelihoods and social support in contexts of social and economic constraint. Health services should thus focus on creating responsive, flexible health service delivery systems that are designed explicitly to support continuity across many shifts in people's everyday life circumstances – including place‐based and support network shifts. Integrated, flexible systems would enable clients to interact continuously with the same health system network rather than stepping between different systems and falling through the gaps.

Increasing efforts to train health workers to be more sensitive to patients’ fluidity will likely foster greater trust and build better relationships with patients that may in turn enable improved collaborative management of fluidity. Similarly, additional resources through counselling and facilitated disclosure processes will likely empower patients to mitigate the influence of social support network fluidity on their care continuum. However, in either case, such interventions are addressing the symptoms and not the underlying cause of fluidity‐related interruptions to the care continuum. We argue for a broader re‐imagining of the health service‐patient interface throughout the health system.

Fundamentally, strategic frameworks for primary health service delivery in southern Africa must integrate responsiveness to granular, everyday shifts in clients’ social and geographic worlds as part of the interpretation of differentiated models of care responsive to experiences of fluidity. One starting point for such a shift requires implementation of existing technologies for ensuring that patient record systems are integrated across health service centres and community‐based health service delivery platforms. Further, clinic forms, patient information management systems, and staff training must be redesigned to work towards the core goal of ensuring continuity of care by offering multiple service options from which patients may choose as suits their fluid life demands. Once these fundamentals are in place there are multiple avenues for optimizing continuity of care. For example, routinely dispensing ART at shifting intervals, according to time periods defined according to patients’ shifting life schedules. Effectively accounting for and working with fluidity, rather than trying to work around it, can potentially create opportunities to tap into wider patient geographies and social networks that support access to continuous care. For example, designing patient indexing systems to see each home as an additional point of contact with that patient.

In HIV policy and implementation, “mobility” has for too long been understood as a barrier to be overcome or as an excuse for poor patient outcomes. In addition, conceptions of mobility that ignore the linked, but discrete, experiential sense of household fluidity, are inadequate to formulate effective HIV service delivery plans in southern Africa. Instead, we propose embracing the resourcefulness of patients and health workers to manage experiences of fluidity to ensure HIV service continuity. The time has come for stability and care continuity through matched fluidity in the health system itself.

## Competing interests

The authors have no competing interests to declare.

## Authors’ contributions

GH led the design of the qualitative cohort data collection process, supervised the analytic team, and led the drafting of this manuscript. HM participated in data collection, led the analysis of observational data, and supported the development of the conceptual heuristic. LdV, RN, JM and MM participated in data collection and led the analysis of interview/discussion data. AT supported drafting the introduction. HA, PB, KS, JH and JS provided expert review to interpret the findings relative to the PopART intervention package and HIV service delivery in Zambia and South Africa. VB led the design of the social science evaluation of HPTN 071 (PopART) and supervised the data collection team in Zambia. LR supported the design of the qualitative cohort data collection process, offered expert oversight for the analytic design and conceptualization of this paper, and provided detailed revisions of the paper itself. All co‐authors participated in the interpretation of data and reviewing the manuscript at different stages.

## Funding

HPTN 071 is sponsored by the National Institute of Allergy and Infectious Diseases (NIAID) under Cooperative Agreements UM1‐AI068619, UM1‐AI068617, and UM1‐AI068613, with funding from the U.S. President's Emergency Plan for AIDS Relief (PEPFAR). Additional funding is provided by the International Initiative for Impact Evaluation (3ie) with support from the Bill & Melinda Gates Foundation, as well as by NIAID, the National Institute on Drug Abuse (NIDA) and the National Institute of Mental Health (NIMH), all part of NIH. The content is solely the responsibility of the authors and does not necessarily represent the official views of the NIAID, NIMH, NIDA, PEPFAR, 3ie, or the Bill & Melinda Gates Foundation.
